# Time to Seroconversion in HIV-Exposed Subjects Carrying Protective versus Non Protective *KIR3DS1/L1 and HLA-B* Genotypes

**DOI:** 10.1371/journal.pone.0110480

**Published:** 2014-10-17

**Authors:** Benjamin J. M. Tallon, Julie Bruneau, Christos M. Tsoukas, Jean-Pierre Routy, Zahra Kiani, Xianming Tan, Nicole F. Bernard

**Affiliations:** 1 Research Institute of the McGill University Health Centre (RI MUHC), Montréal, Québec, Canada; 2 Division of Experimental Medicine, McGill University, Montréal, Québec, Canada; 3 Centre de recherche, Centre Hospitalier de l'Université de Montréal (CRCHUM), Montréal, Québec, Canada; 4 Family Medicine Department, Université de Montréal, Montréal, Québec, Canada; 5 Division of Clinical Immunology and Allergy, McGill University Health Centre, Montréal, Québec, Canada; 6 Immunodeficiency Service and Division of Hematology, Royal Victoria Hospital, McGill University Health Centre, Montréal, Québec, Canada; 7 Biostatistics Core Facility, Research Institute of the McGill University Health Centre, Montréal, Québec, Canada; Karolinska Institutet, Sweden

## Abstract

Natural killer (NK) cells play a role in the clearance of viral infections. Combinations of alleles at the polymorphic *HLA-B* locus and the NK cell surface killer immunoglobulin-like receptor locus *KIR3DL1/S1* have been shown to influence time to AIDS in HIV-infected individuals and risk of seroconversion in HIV exposed seronegative (HESN) subjects. Here, we assessed time to seroconversion or duration of seronegative status in a group of 168 HIV exposed individuals, including 74 seroconverters and 94 HESN based on carriage or not of *KIR3DL1/S1/HLA-B* genotypes previously shown to be associated with protection from infection and/or slow time to AIDS. *KIR3DL1/S1* genotyping was performed by sequence-specific primer polymerase chain reaction using two pairs of specific primers for each locus. The MHC class IB locus was typed to four-position resolution to resolve *Bw4* and *Bw6* alleles and the amino acid present at position 80. *KIR3DL1/S1* heterozygotes became HIV infected significantly faster than *KIR3DS1* homozygotes. Individuals who carried both *KIR3DS1* and Bw4*80I did not remain HIV seronegative longer than those from a control group who were homozygous for *HLA-Bw6* and carried no *HLA-A* locus *Bw4* alleles Subjects who were *h/*y+B*57 showed a trend towards slower time to serconversion than those with other *KIR3DL1* homozygous and *KIR3DL1/S1* heterozygous genotypes. Thus, *KIR3DS1* homozygosity is associated with protection from HIV infection while co-carriage of *KIR3DS1* and Bw4*80I is not. The requirements for protection from HIV infection can differ from those that influence time to AIDS in HIV infected individuals.

## Introduction

Some individuals remain uninfected despite repeated exposures to HIV. Studying these HIV-exposed seronegative (HESN) subjects may provide us with clues as to what constitutes protective immunity to HIV [1] [1].

Elevated natural killer (NK) cell function has been observed in HESN subjects [2]–[4], suggesting that these cells might mediate protection against HIV infection. NK cell function is regulated by the integration of signals received from cell surface activating and inhibitory receptors. Among these is a family of receptors encoded by the killer immunoglobulin-like receptor (KIR) region. Inhibitory KIR (iKIR) interact with HLA-class I molecules. KIRs can have two or three immunoglobulin-like extracellular domains and their function is determined by the composition of their intracellular domain. iKIR have long cytoplasmic tails containing two immunoreceptor-tyrosine-based inhibitory motifs (ITIM) and mediate negative signals upon engaging their ligands. Activating KIR (aKIR) have short intracellular tails with positively-charged transmembrane residues that associate with adaptor molecules such as DAP-12 [5], which bear immunoreceptor tyrosine-based activating motifs (ITAMs); engagement of aKIR leads to NK cell activation through these adaptor molecules [5]. While the ligands for iKIR are well defined, less is known about the ligands for aKIR. To date, the only aKIR with clearly identified ligands are KIR2DS1 [6]–[8] and KIR2DS4 [9]–[11] and both of these bind their ligands with much lower affinity than their inhibitory counterparts.

The *KIR3DL1/S1* locus has been the subject of several disease association studies. Of the 15 genes and 2 pseudogenes within the KIR genetic region, *KIR3DL1* is the most polymorphic, with more than 70 inhibitory and 13 activating alleles [12]. The locus is unique among KIR genes in that it codes for both inhibitory (KIR3DL1) and activating (KIR3DS1) receptors. The genes coding for KIR are inherited in haplotypes, nearly all of which contain either a *KIR3DL1* or *KIR3DS1* allele, though haplotypes lacking both genes have been reported as well [13], [14]. KIR3DL1 interacts with HLA-Bw4 and binds with the highest affinity to HLA-Bw4 antigens with an isoleucine at position 80 of the heavy chain (Bw4*80I) [15]. KIR3DS1 is presumed to bind to Bw4 as well, though this has not been formally proven [5], [16], [17]. Despite this, carriage of *KIR3DS1* with its putative ligand associates with delayed HIV-disease progression [18]. In HESN, carriage of *KIR3DS1* without its ligand is linked to protection from infection [19], [20] and when co-carried with its putative ligand with increased risk of HIV acquisition [21]. The co-carriage of high expression *KIR3DL1* genotypes with HLA-B*57 (*h/*y+B*57) is associated with both slow HIV disease progression and protection from infection [22], [23]. Additionally, functional evidence suggests that KIR3DS1^+^ NK cells from individuals who carry Bw4*80I and *h/*y+B*57 carriers are more efficient at inhibiting HIV replication in autologous CD4^+^ T cells *in vitro* than NK cells from subjects who have the receptor or the ligand only or neither [24], [25]. Bw6 antigens do not act as ligands for either KIR3DL1 or KIR3DS1 receptors [26].

NK cells from a Vietnamese cohort of HESN injection drug users (IDU) are more cytolytic *ex vivo* in response to stimulation with an HLA-null cell line than NK cells from HIV-negative IDU who eventually seroconverted, suggesting an innate difference in the ability of NK cells from these two study groups to respond to targets [2]. We previously demonstrated a significantly higher frequency of *KIR3DS1* homozygous and *h/*y+B*57 genotypes in HESN subjects compared to HIV-susceptible subjects enrolled in a primary infection (PI) cohort [19], [23]. In this report we performed a time-to-outcome analysis in HIV-exposed individuals stratified according to the three generic *KIR3DL1/S1* genotypes, presence of *KIR3DS1* and Bw4*80I versus at least one copy of *KIR3DL1* and no *Bw4* alleles or presence of *h/*y+B*57 carrier status versus other *KIR3DL1* homozygous and *KIR3DL1/S1* heterozygous genotypes to assess the impact of these genotypes on the time to seroconversion.

## Methods

### Ethics Statement

This study, which used cells as a source of DNA and clinical follow up information from HIV-infected and uninfected subjects, was conducted in compliance with the principles included in the Declaration of Helsinki. This study received approval from the Institutional Review Board of the McGill University Health Center and Centre Hospitalier de l’Université de Montréal -Research Center, Montreal, Canada. All blood donors provided written informed consent for their participation in the study, for the use of their blood for the isolation of peripheral blood mononuclear cells (PBMC) and the use of their PBMC to prepare Epstein-Barr virus (EBV)-transformed B cell lines. All of the EBV lines used to isolate DNA for the HLA and KIR allotyping presented in this manuscript were prepared in house. The research conformed to ethical guidelines of all the authors’ institutions.

### Study Populations

This study included 168 individuals followed longitudinally, of which 94 remained uninfected and met the criteria for classification as HESN and 74 seroconverted to HIV (SC). HESN were recruited from the St. Luc cohort, a prospective cohort of active HIV-negative injection drug users (IDU) at high risk for HIV acquisition (n = 75) [27], and among HIV-negative partners of serodiscordant couples followed in medical clinics in Montreal (n = 19) [28]. SC were recruited from IDU initially followed in the St. Luc cohort (n = 71) [27] and from previously HIV-negative partners in serodiscordant relationships (n = 3). HESN subjects were followed longitudinally every six months. Follow-up included assessment of the frequency of high-risk behaviour for HIV acquisition, blood draws and monitoring of HIV serostatus. All HESN subjects maintained a negative HIV enzyme immunoassay (HIV EIA) test despite at least five reported HIV exposures. Parenteral exposure was defined as sharing needles with known HIV-infected partners and mucosal exposure was defined as unprotected sex with a known HIV-infected partner. In the St Luc cohort needle sharing with HIV-infected persons and cocaine use were previously shown to be predictors of HIV seroconversion [29]. None of the HESN subjects were *CCR5Δ32* homozygotes, a genotype known to confer resistance to HIV infection [30], [31]. The median (range) age in years for HESN and SC was 47 (23, 63) and 39 (26, 54), respectively and 45 (23, 62) for the entire study population. Follow-up (FUP) years for HESN and SC were 13 (0.2, 25.9) and 9 (0.06, 17.8), respectively.

### Genotyping

Genomic DNA was extracted from PBMC or EBV-transformed B cell lines using a QIAamp DNA blood kit (QIAGEN, Inc., Mississauga, Ontario, Canada). *KIR3DL1/S1* genotyping was performed by polymerase chain reaction (PCR) with sequence-specific primers based on previously published studies [18], [32], [33]. Two pairs of primers specific for *KIR3DL1* or *KIR3DS1* sequences were used to amplify these allele families. Primers for *NKG2A* were also included as positive controls. *KIR3DS1* homozygosity was defined as presence of *KIR3DS1* in the absence of *KIR3DL1*. Heterozygosity at this locus was defined as presence of both *KIR3DL1* and *KIR3DS1*. *KIR3DL1* homozygosity was defined as the presence of *KIR3DL1* in the absence of *KIR3DS1*. Copy-number variation (CNV) exists at this locus that can result in either the duplication or deletion of a copy of *KIR3DL1* or *KIR3DS1*, which can have functional implications regarding the ability of NK cells to inhibit viral replication *in vitro* [34]. Thus, subjects were assessed for CNV at the *KIR3DL1/S1* locus [34]. *KIR3DL1* homozygotes and *KIR3DL1/S1* heterozygotes were *KIR3DL1* subtyped at the allele level by gene sequencing as previously described [23]. Subjects were typed for HLA class I using the line probe assay (Innogenetics Inc, Alpharetta, Georgia, USA) or by sequencing (Atria Genetics, South San Francisco, California, USA) to resolve the assignment of *HLA-B* alleles to the Bw4 or Bw6 public specificities based on amino acids at positions 77 to 83 [35].

### Determining time to seroconversion

For each subject, time 0 was operationally defined as the date of the first reported sharing episode for IDU and of the first sexual contact of the HESN subjects with their seropositive partner in serodiscordant couples. For some subjects, this “first sharing” date was before HIV infection reached a prevalence of 10% in the IDU population in Montreal; all reported dates of first sharing were moved to September 1^st^ 1988 if they occurred before this date [36]. Event time was defined as the difference between the estimated date of seroconversion and time 0. Subjects who did not seroconvert were censored at the time of their last study visit within six months of a documented exposure. For SC, the date of seroconversion was determined using the algorithm proposed by the Acute HIV Infection Early Disease Research Program sponsored by the National Institutes of Health [37], [38]. Briefly, the estimated date of infection was obtained by subtracting 14 days from the date of a positive HIV viral load (VL) test or p24 antigen assay available on the same day as a negative HIV EIA test or the date of onset of symptoms of an acute retroviral syndrome, or 35 days from the date of first indeterminate Western blot. In addition, information obtained from questionnaires addressing the timing of high-risk behaviour for HIV transmission was taken into account in assigning a date of infection when consistent with biological tests. The results of a less sensitive HIV EIA (LS-EIA) which identifies infected subjects within a window period of 170 days from infection (95% confidence interval 162–183 days) were used to confirm the estimated date of infection [39]. In some cases where such information was not available the mid-point between the last negative HIV EIA and the first positive HIV EIA was used as the presumed date of infection. For each HESN subject, the date of censoring was taken as the date of the subject's last available seronegative visit following a six month time interval in which HIV exposure had occurred.

### Statistical Analysis

GraphPad InStat v. 3.10, GraphPad Prism v.5.04 and SAS v. 9.2 for Windows were used for statistical analysis and graphical presentation. A Mann-Whitney test was used to compare behavioural parameters between HESN IDU and SC subjects. A Wald test was used to compare the difference in time to seroconversion between subjects with different generic *KIR3DL1/S1* genotypes, those who were KIR3DS1+Bw4*80I versus *Bw6* homozygotes, and those who were *h/*y+B*57 versus carrying other *KIR3DL1* homozygous and *KIR3DL1/S1* heterozygous genotypes. In addition, a Cox regression model adjusting age and gender was applied to verify the impact of *KIR3DL1/S1* genotypes on time-to-seroconversion. We accounted for late entry (or left truncation) [40], [41] when we conducted the above Wald test and Cox regression analysis by including the entry time (time elapsed from the “first sharing” to study entry) in the analysis. Accounting for late entry is necessary in this study because subjects who were HIV positive at study entry were (and should be) excluded from study, and resulted in a situation called *late entry* or *left truncation* in survival analysis. A p-value of less than 0.05 was considered significant.

## Results

### Study population characteristics

Study subjects were drawn from the same geographic area (Montreal, Quebec, Canada) and were approximately 95% Caucasian and predominantly male (see Table 1). To determine if patterns of drug use differed between IDU in the HESN and SC populations, we compared age at event (seroconversion or censoring), age at first sharing, and duration of sharing. HESN IDU subjects at censoring versus SC at event had a higher median age (47 versus 39 yrs, p<.0001, Mann-Whitney test), and duration of sharing (median period of sharing, 13 yrs, p<.0001, Mann-Whitney test).

**Table 1:** Population Characteristics.

	HESN (n = 94)		SC (n = 74)	
Characteristic	IDU (%) [n = 75]	SE^a^ (%) [n = 19]	IDU (%) [n = 71]	SE (%) [n = 3]
Male	60 (80.0)	9 (47.4)	62 (87.3)	1 (33.3)
Female	14 (18.7)	10 (52.6)	7 (9.9)	2 (66.6)
N/A	1 (1.3)	0 (0)	2 (2.8)	0 (0)
Ethnicity				
Caucasian	69 (92)	16 (84.2)	71 (100.0)	3 (100.0)
Black	2 (2.7)	0 (0)	0 (0)	0 (0)
Asian	0 (0)	1 (5.3)	0 (0)	0 (0)
Native	1 (1.3)	2 (10.5)	0 (0)	0 (0)
Other	3 (4.0)	0 (0)	0 (0)	0 (0)

Abbreviations: HESN, HIV-exposed seronegative; SC, seroconvertors; IDU, injection drug users; SE, sexually exposed; N/A, information not available.

a Sexual exposure includes men who have sex with men and heterosexual exposure in both the male-to-female and female-to-male direction.

We previously reported a higher frequency of *KIR3DS1* homozygosity in HESN compared to HIV-susceptible subjects [19]. In this study *KIR3DS1* homozygous HESN and SC had a similar age at event and age at first sharing though the duration of exposure was longer in HESN than in SC IDU subjects (median period of sharing, 13 yrs versus 9 yrs, p = 0.0311, Mann-Whitney test), In summary, maintenance of HIV seronegative status in HESN IDU subjects could not be explained by a shorter duration of exposure to HIV than that seen in SC IDU. So too, subjects carrying the “protective” *KIR3DS1* homozygous genotype did not remain seronegative simply because they were HIV exposed for a shorter time than SC.

### Time to seroconversion in groups categorized by KIR3DL1/S1 genotype

**Figure 1:**
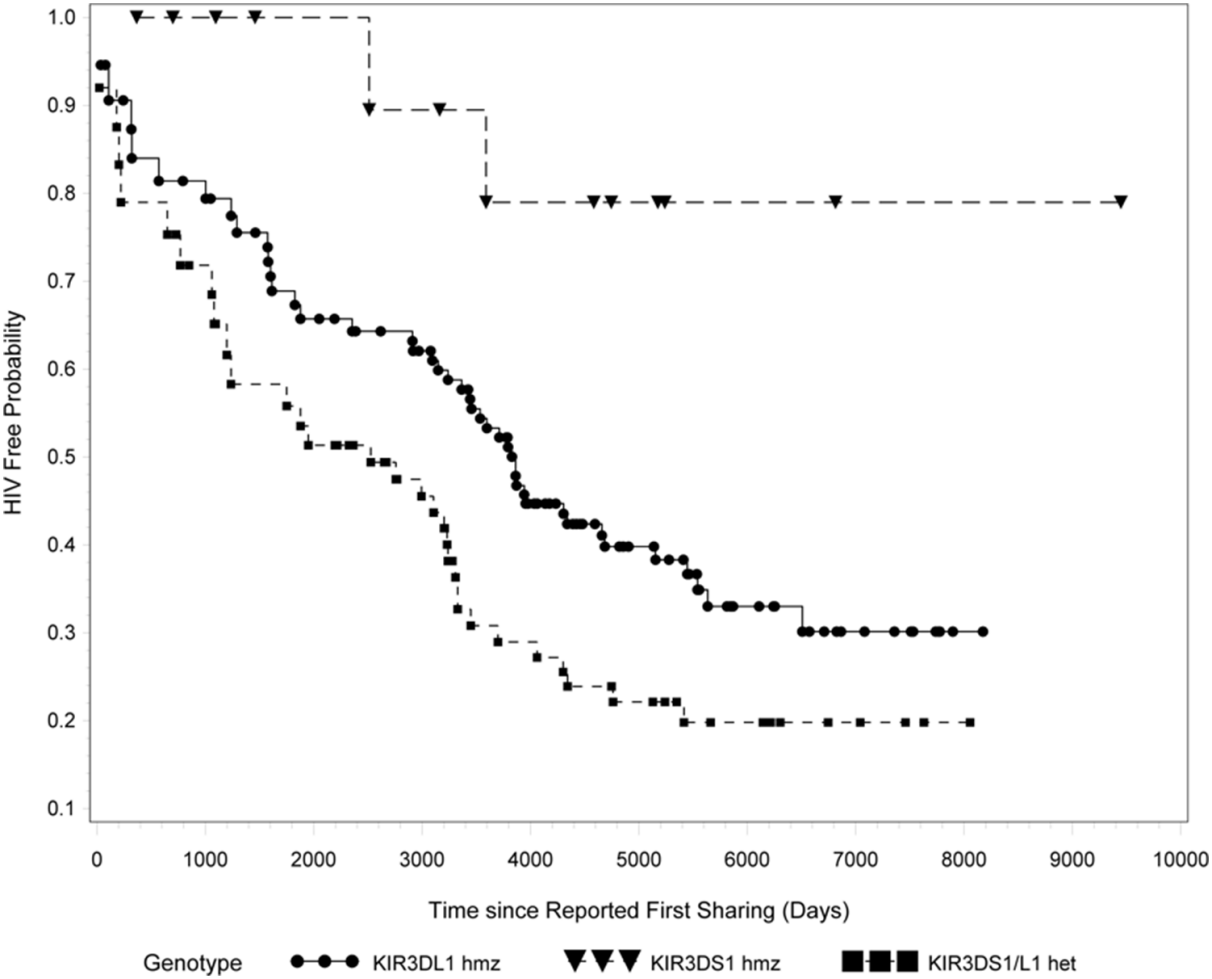
Time-to-event based on carriage of three generic KIR3DL1/S1 genotypes. All HIV exposed *KIR3DS1* homozygotes (n = 14, 12 HIV exposed seronegative [HESN] and 2 seroconverters [SC]), *KIR3DL1* homozygotes (n = 96, 55 HESN and 41 SC) and *KIR3DL1/S1* heterozygotes (n = 58, 27 HESN and 31 SC) were compared for time to event (either seroconversion or censoring). P-value was calculated using a Wald test adjusted for late entry.

To investigate whether the *KIR3DL1/S1* genotypes conferred a differential effect on time to seroconversion we performed a time-to-event analysis, stratifying subjects according to *KIR3DL1/S1* genotypes. Table S1 shows the characteristics of the study population, including the gender, ethnic origin, risk category and *KIR3DL1/S1* genotype of each HESN and SC. Table S1 also includes the date of first sharing, date of censoring for HESN as well as the duration of FUP for HESN. Table S2 lists the date of seroconversion and duration of HIV negative status for SC. The number of SC per genotype group was 41 of 96 (42.7%) *KIR3DL1* homozygotes, 31 of 58 (53.4%) *KIR3DL1/S1* heterozygotes and 2 of 14 (14.3%) *KIR3DS1* homozygotes. Carriage of the *KIR3DL1/S1* heterozygous genotype was associated with a significantly faster time to seroconversion compared to carriage of the *KIR3DS1* homozygous genotype (p = .0146, Wald test adjusted for late-entry) (Figure 1). Subjects who were *KIR3DL1* homozygotes also had a faster time to seroconversion than *KIR3DS1* homozygotes, though this did not reach statistical significance (p = .0635, Wald test adjusted for late entry) (Figure 1). Comparison of time to seroconversion between *KIR3DS1* homozygotes and *KIR3DL1/S1* heterozygotes remained significant when the study population was limited to those exposed through IDU (n = 146) (p = 0.04, not shown) and only Caucasian IDU subjects (n = 140) (p = 0.04, not shown). The significant difference in time to seroconversion between *KIR3DS1* homozygous and *KIR3DL1/S1* heterozygous subjects remained after controlling for age and gender (Table 2).

**Table 2:** Statistics for Survival Analysis.

	All Subjects (n = 163)		IDU Only (n = 146)		IDU Caucasian Only (n = 140)	
Parameter	Hazard Ratio	p	Hazard Ratio	p	Hazard Ratio	p
KIR3DL1 Hmz	3.439	0.0899	2.56	0.1999	2.806	0.1597
KIR3DL1/S1 Het	5.34	0.0299	4.261	0.05	4.395	0.0457
Age	0.982	0.3426	0.979	0.3081	0.98	0.3415
Gender	0.709	0.347	0.947	0.8921	1.01	0.9802

All hazard ratios and p values are reported as compared to the *KIR3DS1* homozygous genotype. Five subjects were excluded due to lack of information about gender or date of birth. P values calculated using a Wald test.

The *KIR3DL1/S1* locus is subject to CNV that can include duplication or deletion of the *KIR3DL1* and *KIR3DS1* genes [34]. We performed a CNV screen on 164 subjects of the initial 168 subjects (four were excluded due to lack of CNV information). A total of 10 subjects had a CNV at the *KIR3DL1/S1* locus (see Table S3 for characterization of subjects with CNV). When these 10 individuals were excluded from analysis, carriage the *KIR3DL1/S1* heterozygous genotype was still associated with faster time to seroconversion compared to carriage of the *KIR3DS1* homozygous genotype (p = 0.03) (not shown).

### Time to seroconversion in carriers of the KIR3DL1*h/*y+B*57 versus other KIR3DL1/S1/HLA-B genotypes

Table S4 shows the HLA-A and B alleles carried by all HESN and SC for whom HLA types were available and indicates which subjects carried the *h/*y+B*57 KIR/HLA combination and which among the *KIR3DL1* homozygotes and *KIR3DL1/S1* heterozygotes did not. We performed a time-to-event analysis, comparing subjects with the *h/*y+B*57 genotype with all other *KIR3DL1* homozygotes and *KIR3DL1/S1* heterozygotes. *KIR3DS1* homozygotes were excluded from this analyses based on their having a slower time to seroconversion. For this analysis the number of SC was 71 of 146 (48.60%) *KIR3DL1* homozygotes and *KIR3DL1/S1* heterozygotes and 1 of 7 (14.29%) *h/*y+B*57 subjects. Those who were not *h/*y+B*57 carriers showed a non-significant trend towards a faster time to seroconversion compared to carriers of *h/*y+B*57 (p = .12,) (Figure S1). The trend was maintained if analyses were limited to only IDU (n = 138) or only Caucasian IDU (n = 131) (not shown).

### Co-carriage of KIR3DS1 and Bw4*80I does not associate with protection from infection

**Figure 2:**
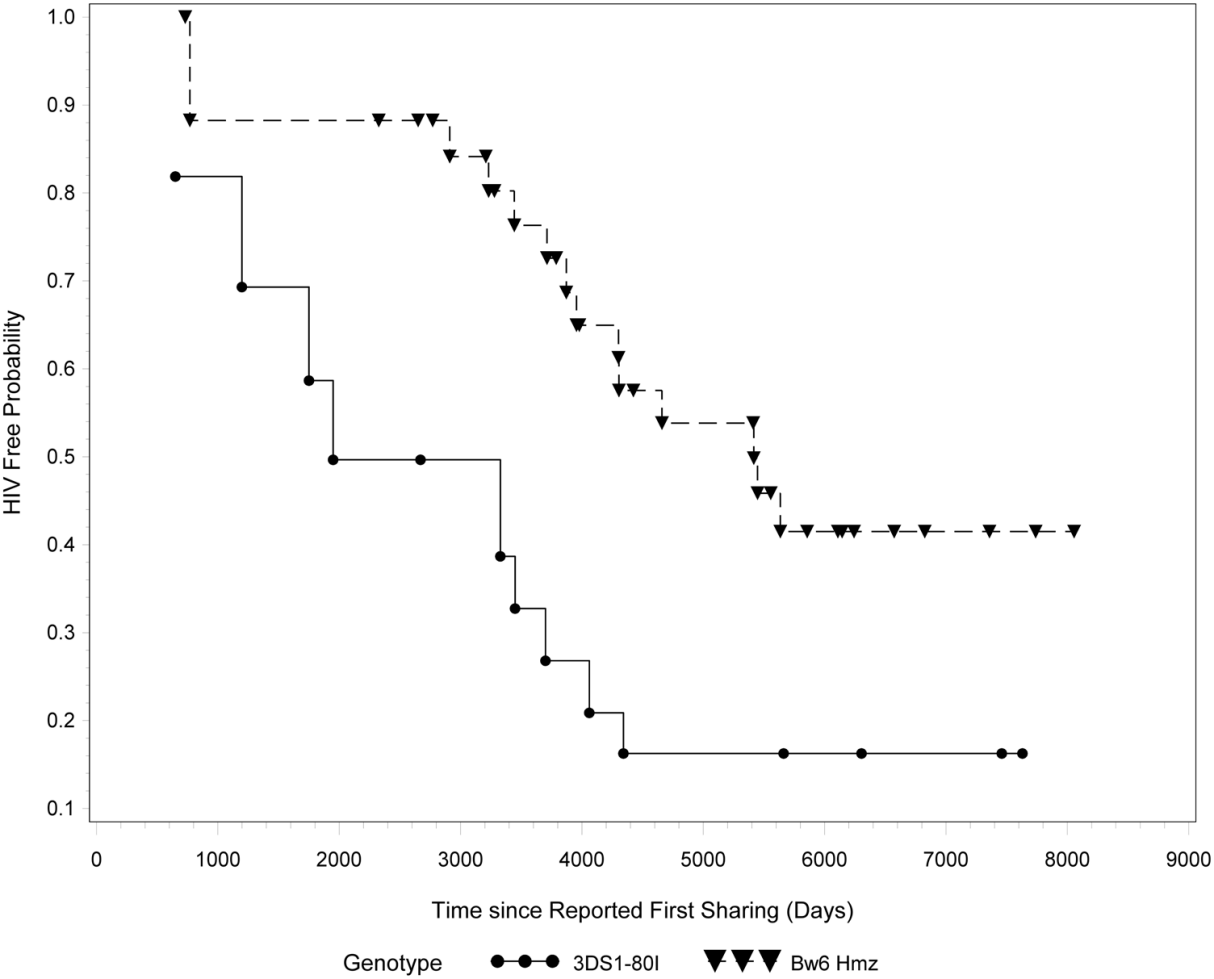
Time to event based on carriage of KIR3DS1+Bw4*80I or Bw6 homozygosity. HIV exposed *Bw6* homozygotes (n = 34, 21 HESN and 13 SC) and carriers of the KIR3DS1+Bw4*80I genotype (n = 15, 5 HESN and 10 SC) were compared for time to event (either seroconversion or censoring). P-value was calculated using a Wald test adjusted for late entry.

Co-carriage of *KIR3DS1* and Bw4*80I alleles has been implicated in slower progression to AIDS following HIV infection [18]. KIR3DS1^+^ NK cells from individuals who carried Bw4*80I were more efficient at inhibiting viral replication *in vitro* than those who did not carry this putative receptor-ligand combination [42]. We questioned whether this combination was also associated with protection from infection in our cohort. Table S4 lists the individuals included in this analysis. *KIR3DL1/S1* heterozygotes who carried at least one copy Bw4*80I at the *HLA-B* locus (n = 15) were compared to *Bw6* homozygotes with no *Bw4* alleles at the *HLA-A* locus (n = 34). Carriers of *KIR3DS1* and Bw4*80I became infected faster than *Bw6* homozygotes (p = 0.036, Wald test) if the KIR3DS1+Bw4*80I group was expanded to include those with an Bw4*80I allele at the *HLA-A* locus as well (A*23, A*24, A*25 or A*32) (n = 28) [43]. They also seroconverted faster than *Bw6* homozygotes though the difference did not achieve statistical significance (p = 0.08, Wald test) (not shown) (Figure 2).

## Discussion

In this study we report that HIV exposed carriers of the *KIR3DL1/S1* heterozygous genotype seroconvert faster than those carrying *KIR3DS1* without *KIR3DL1.* This finding was significant if all study subjects, only IDU or only Caucasian IDU were included in the analysis. The observation remained significant if 10 individuals exhibiting CNV at the *KIR3DL1/S1* locus were excluded from the analysis. Carriers of a second genotype previously associated with protection from infection, i.e. *h/*y+B*57 showed a non-significant trend towards slower time to seroconversion compared with carriers of other *KIR3DL1* homozygous and *KIR3DL1/S1* heterozygous genotypes. HIV exposed subjects carrying *KIR3DS1* and Bw4*80I, a combination shown previously to associate with delayed progression to AIDS, was not protective in terms of time to seroconversion and may even be associated with a faster time to seroconversion.

The population studied here included persistently seronegative individuals classified as HESN who had at least five documented exposures to HIV, and HIV-susceptible SC. Between-group differences in behavioural patterns for the IDU subset of the study population could not account for maintenance of seronegativity in HESN subjects since HESN drug use patterns were either similar to or supported higher exposure levels than those in the SC group. This was also the case for comparisons of HESN IDU who had at least one copy of *KIR3DS1* without *KIR3DL1* to all SC.

Survival analysis showed that individuals carrying the *KIR3DS1* homozygous genotype had a slower time to event compared to those carrying the other generic genotypes at this locus. Notably, this effect was significant when *KIR3DS1* homozygotes were compared to *KIR3DL1/S1* heterozygotes. (p = .0146, Wald test, Figure 1 and Table 2). These results suggest that carriage of *KIR3DL1* together with *KIR3DS1* is not beneficial with respect to protection from HIV infection. Of the 14 subjects in the *KIR3DS1* homozygote group, one exhibited copy number variation with a single *KIR3DS1* allele. Differences in time to seroconversion between *KIR3DS1* homozygotes and *KIR3DL1/S1* heterozygotes remained significant even if this individual was excluded. The slight protective effect conferred by *KIR3DL1* homozygosity over *KIR3DL1/S1* heterozygosity at this locus (Figure 1) may in part be due to the possibility the *KIR3DL1* homozygous group includes individuals positive for the protective *h/*y+B*57 genotype [44].

The *h/*y+B*57 combination has been reported to associate with delayed progression to AIDS [45]. Thus, we were interested in knowing whether HIV exposed carriers of this genotype were also protected at the level of time to seroconversion. Although we previously found a higher proportion HESN than HIV susceptible of individuals carrying *h/*y+B*57 [23] the small number of persons with this KIR/HLA combination and pre-infection longitudinal follow-up precluded making a firm conclusion on whether this genotype supported a slower time to infection.

We found no evidence that carriage of at least one copy of *KIR3DS1* with Bw4*80I was protective (Figure 2), despite its reported association with delayed HIV disease progression [18]. Time to event analyses performed on a prospective cohort in Tanzania found that carriers of this genotype combination showed increased HIV acquisition compared to carriers of other *KIR3DL1/S1 HLA-B* genotypes [21]. Together these results suggest that the mechanisms that these genotypes influence with respect to delayed progression to AIDS differ from those that contribute to protection from HIV infection.

What these mechanisms may be are not known at present. Alter et al. have shown that NK cells from carriers of the KIR3DS1+Bw4*80I inhibit HIV replication in autologous HIV infected CD4 cells [46]. On the other hand there is no evidence that KIR3DS1 and HLA-Bw4*80I can directly interact [47], [48]. If they did it would be expected that the function of these KIR3DS1 positive NK cells would be tuned down rather than up since KIR3DS1 is an activating NK cell receptor [49], [50]. Therefore, the anti-HIV activity of NK cells from carriers of this KIR/HLA genotype probably depends on the presence of HIV. One possibility is that HIV infection induces ligands for activating NK receptors, which in turn stimulate NK cells for anti-HIV activities such as secretion of chemokines that block HIV entry [25], [51]. If this is the case it may explain why NK cell from carriers of *KIR3DS1* and HLA-Bw4*80I protect in the context of HIV infection but do not at the level of protection from HIV infection, where there may be few HIV infected cells present at very early infection. The mechanisms underlying this phenomenon merit further exploration.

Though *KIR3DS1* homozygosity has been previously identified as a marker of protection from HIV infection, this is, to the best of our knowledge, the first longitudinal study of this genotype in a cohort of HESN and seroconvertors. Our results indicate that compared to *KIR3DS1* homozygotes, repeatedly HIV-exposed carriers of other *KIR3DL1/S1* genotypes exhibit a faster time to infection.

## Supporting Information

Figure S1 Time-to-event based on carriage of the *h/*y+B*57 versus KIR3DL1 homozygous and KIR3DL1/S1 heterozygous genotypes. All HIV exposed *h/*y+B*57 carriers (n = 7, 6 HESN and 1 SC), *KIR3DL1* homozygotes and *KIR3DL1/S1* heterozygotes (n = 146, 75 HESN and 71 SC) were compared for time to event (either seroconversion or censoring). P-value was calculated using a Wald test adjusted for late entry.
(TIF)

Table S1 **Study population characteristics.**
(DOCX)

Table S2 **Study population characteristics and** ***KIR3DL1/S1*** **genotype for Seroconverters.**
(DOCX)

Table S3 **Genotype Group of Subjects with Copy Number Variation for** ***KIR3DL1/S1.***
(DOCX)

Table S4 **Study population HLA types and KIR/HLA genotype categoriess used in analyses.**
(DOCX)
