# Supplementary figures and images for: Time to Seroconversion in HIV-Exposed Subjects Carrying Protective versus Non Protective KIR3DS1/L1 and HLA-B Genotypes
Source: PLoS One. 2014 Oct 17;9(10):e110480. doi: 10.1371/journal.pone.0110480 (PMC4201542; doi:10.1371/journal.pone.0110480)

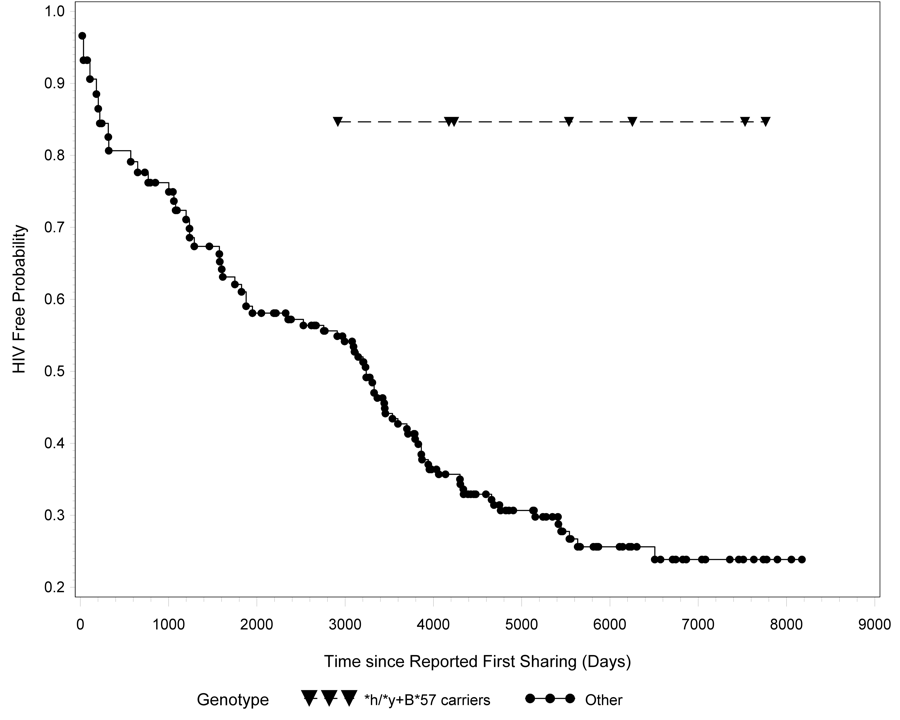

Supplement: Figure S1 — Time-to-event based on carriage of the *h/*y+B*57 versus KIR3DL1 homozygous and KIR3DL1/S1 heterozygous genotypes. All HIV exposed *h/*y+B*57 carriers (n = 7, 6 HESN and 1 SC), KIR3DL1 homozygotes and KIR3DL1/S1 heterozygotes (n = 146, 75 HESN and 71 SC) were compared for time to event (either seroconversion or censoring). P-value was calculated using a Wald test adjusted for late entry. (TIF) [file pone.0110480.s001.tif]
