# Supplementary material for: Time to Seroconversion in HIV-Exposed Subjects Carrying Protective versus Non Protective KIR3DS1/L1 and HLA-B Genotypes
Source: PLoS One. 2014 Oct 17;9(10):e110480. doi: 10.1371/journal.pone.0110480 (PMC4201542; doi:10.1371/journal.pone.0110480)
Supplement: Table S1 — Study population characteristics. (DOCX) [file pone.0110480.s002.docx]

**Table S1. Study population characteristics.**

| **ID** | **M/F^1^** | **Race^2^** | **Risk^3^** | **Serostatus^4^** | **3DL1/S1 Genotype^5^** | **First Sharing^6^** | **Censoring Date HESN^7^** | **Duration of HIV negative status (HESN)^8^** |
| --- | --- | --- | --- | --- | --- | --- | --- | --- |
| H_ANN | F | C | IDU | HESN | 3DL1hmz | 09-Jul-07 | 25-Sep-07 | 78 |
| X_BDE | M | C | IDU | HESN | 3DL1hmz | 15-Sep-98 | 16-Sep-02 | 1462 |
| M_LRB | M | C | IDU | HESN | 3DL1hmz | 01-Aug-95 | 13-Mar-01 | 2051 |
| M_HAS | M | C | IDU | HESN | 3DL1hmz | 01-Sep-88 | 06-Jan-99 | 3779 |
| X_ADT | F | C | IDU | HESN | 3DL1hmz | 15-Jul-98 | 19-Dec-06 | 3079 |
| S_RCK | M | C | IDU | HESN | 3DL1hmz | 05-Sep-00 | 21-Jan-10 | 3425 |
| X_ADX | M | C | IDU | HESN | 3DL1hmz | 01-Sep-88 | 12-Oct-00 | 4424 |
| M_MMZ | M | C | IDU | HESN | 3DL1hmz | 01-Sep-88 | 16-Nov-00 | 4459 |
| M_EAR | M | C | IDU | HESN | 3DL1hmz | 01-Sep-88 | 07-Dec-00 | 4480 |
| X_JCA | M | C | IDU | HESN | 3DL1hmz | 15-Jan-92 | 30-May-02 | 3788 |
| X_FHV | M | C | IDU | HESN | 3DL1hmz | 15-Dec-98 | 05-Nov-09 | 3978 |
| M_JEF | M | C | IDU | HESN | 3DL1hmz | 01-Sep-88 | 05-Feb-02 | 4905 |
| X_MIW | M | C | IDU | HESN | 3DL1hmz | 15-Jul-92 | 12-Nov-03 | 4137 |
| X_EMX | M | C | IDU | HESN | 3DL1hmz | 15-Sep-96 | 20-Feb-08 | 4175 |
| X_ITL | M | C | IDU | HESN | 3DL1hmz | 15-Jul-92 | 17-Feb-04 | 4234 |
| M_LHE | F | C | IDU | HESN | 3DL1hmz | 01-Sep-88 | 27-Sep-02 | 5139 |
| X_DLH | F | C | IDU | HESN | 3DL1hmz | 15-Jul-95 | 24-Jul-07 | 4392 |
| M_JZC | M | C | IDU | HESN | 3DL1hmz | 01-Sep-88 | 13-Feb-03 | 5278 |
| M_LZC | M | C | IDU | HESN | 3DL1hmz | 01-Sep-88 | 26-Jun-03 | 5411 |
| X_BWC | M | C | IDU | HESN | 3DL1hmz | 15-Apr-95 | 25-Jun-08 | 4820 |
| X_DFN | F | C | IDU | HESN | 3DL1hmz | 15-Jul-92 | 31-Oct-05 | 4856 |
| X_LYZW | M | C | IDU | HESN | 3DL1hmz | 15-Jul-95 | 18-Dec-08 | 4905 |
| M_AIB | M | C | IDU | HESN | 3DL1hmz | 01-Sep-88 | 24-May-05 | 6109 |
| M_BCR | M | C | IDU | HESN | 3DL1hmz | 15-Jul-92 | 01-Jul-07 | 5464 |
| X_GCA | M | C | IDU | HESN | 3DL1hmz | 15-Jul-92 | 10-Sep-07 | 5535 |
| M_GEF | M | C | IDU | HESN | 3DL1hmz | 01-Jun-94 | 17-Aug-09 | 5556 |
| X_FLI | M | C | IDU | HESN | 3DL1hmz | 01-Sep-88 | 31-Aug-06 | 6573 |
| M_NSZ | M | C | IDU | HESN | 3DL1hmz | 15-May-94 | 14-Apr-10 | 5813 |
| X_AGI | M | C | IDU | HESN | 3DL1hmz | 15-Jul-95 | 27-Jul-11 | 5856 |
| X_CDC | M | C | IDU | HESN | 3DL1hmz | 15-Jul-95 | 09-Aug-11 | 5869 |
| M_BMF | M | C | IDU | HESN | 3DL1hmz | 01-Sep-88 | 10-May-07 | 6825 |
| X_NSX | M | C | IDU | HESN | 3DL1hmz | 15-Jul-89 | 28-Nov-07 | 6710 |
| X_EHD | M | C | IDU | HESN | 3DL1hmz | 01-Sep-88 | 22-Jan-08 | 7082 |
| M_LEG | M | C | IDU | HESN | 3DL1hmz | 15-Jul-92 | 17-Aug-09 | 6242 |
| X_IBL | M | C | IDU | HESN | 3DL1hmz | 15-Jan-89 | 09-Mar-09 | 7358 |
| M_CGX | M | C | IDU | HESN | 3DL1hmz | 01-Sep-88 | 26-Mar-09 | 7511 |
| X_YDKC | M | C | IDU | HESN | 3DL1hmz | 01-Sep-88 | 09-Nov-09 | 7739 |
| M_EHF | M | C | IDU | HESN | 3DL1hmz | 01-Sep-88 | 04-Dec-09 | 7764 |
| X_RHG | M | C | IDU | HESN | 3DL1hmz | 01-Sep-88 | 16-Dec-09 | 7776 |
| H_ALA | M | C | IDU | HESN | 3DL1hmz | 01-Sep-88 | 16-Apr-10 | 7897 |
| X_LTG | M | C | IDU | HESN | 3DL1hmz | 01-Sep-88 | 19-Jan-11 | 8175 |
| M_AMC | F | C | IDU | HESN | 3DL1hmz | 01-Sep-88 | 19-Jun-07 | 6865 |
| M_AOD | F | C | IDU | HESN | 3DL1hmz | 01-Sep-88 | 14-Apr-09 | 7530 |
| HESN_DC_001 | F | A | HS | HESN | 3DL1hmz | 01-Jan-97 | 01-Sep-97 | 243 |
| HESN_DC_002 | F | C | HS | HESN | 3DL1hmz | 01-Feb-82 | 01-Feb-98 | 5844 |
| HESN_DC_003 | F | C | HS | HESN | 3DL1hmz | 01-Dec-94 | 01-Feb-97 | 793 |
| HESN_DC_004 | F | C | HS | HESN | 3DL1hmz | 01-Jul-83 | 01-Jul-89 | 2192 |
| HESN_DC_005 | F | C | HS | HESN | 3DL1hmz | 01-May-93 | 01-Jul-00 | 2618 |
| HESN_DC_006 | M | C | MSM | HESN | 3DL1hmz | 15-Nov-02 | 29-May-09 | 2387 |
| HESN_DC_021 | M | C | MSM | HESN | 3DL1hmz | 15-Jul-02 | 01-Sep-10 | 2970 |
| M_LDN | M | Other | IDU | HESN | 3DL1hmz | 01-Jan-91 | 15-Feb-08 | 6254 |
| X_CFV | M | Black | IDU | HESN | 3DL1hmz | 15-Jan-99 | 29-Nov-01 | 1049 |
| X_DHZ | M | Other | IDU | HESN | 3DL1hmz | 01-Jan-91 | 18-Jan-02 | 4035 |
| X_HPJ | M | Black | IDU | HESN | 3DL1hmz | 15-Dec-96 | 15-Jul-09 | 4595 |
| X_IIM | F | Other | IDU | HESN | 3DL1hmz | 01-Jan-91 | 14-Feb-02 | 4062 |
| X_BDYB | M | C | IDU | SC | 3DL1hmz | 13-Jun-01 |  |  |
| M_MML | M | C | IDU | SC | 3DL1hmz | 15-Sep-95 |  |  |
| X_HVK | M | C | IDU | SC | 3DL1hmz | 15-Jan-94 |  |  |
| X_RAI | M | C | IDU | SC | 3DL1hmz | 15-Jan-98 |  |  |
| X_EKL | M | C | IDU | SC | 3DL1hmz | 15-Jul-95 |  |  |
| M_MVB | M | C | IDU | SC | 3DL1hmz | 15-Jun-92 |  |  |
| M_DFH | M | C | IDU | SC | 3DL1hmz | 01-Sep-88 |  |  |
| X_FMH | M | C | IDU | SC | 3DL1hmz | 01-Sep-88 |  |  |
| X_KDX | M | C | IDU | SC | 3DL1hmz | 01-Sep-88 |  |  |
| X_MFM | M | C | IDU | SC | 3DL1hmz | 15-Jul-96 |  |  |
| M_FWB | F | C | IDU | SC | 3DL1hmz | 15-Dec-93 |  |  |
| M_PVN | M | C | IDU | SC | 3DL1hmz | 01-Sep-88 |  |  |
| M_KJE | M | C | IDU | SC | 3DL1hmz | 01-Sep-88 |  |  |
| M_NFC | M | C | IDU | SC | 3DL1hmz | 01-Sep-88 |  |  |
| M_KTC | F | C | IDU | SC | 3DL1hmz | 01-Sep-88 |  |  |
| X_LMA | M | C | IDU | SC | 3DL1hmz | 01-Sep-88 |  |  |
| X_PCV | M | C | IDU | SC | 3DL1hmz | 01-Sep-88 |  |  |
| M_LII | M | C | IDU | SC | 3DL1hmz | 15-Sep-93 |  |  |
| X_NVJ | M | C | IDU | SC | 3DL1hmz | 15-Jan-98 |  |  |
| X_GKT | M | C | IDU | SC | 3DL1hmz | 01-Sep-88 |  |  |
| X_ZMYK | M | C | IDU | SC | 3DL1hmz | 15-Jul-96 |  |  |
| M_LFW | M | C | IDU | SC | 3DL1hmz | 01-Sep-88 |  |  |
| X_IRM | F | C | IDU | SC | 3DL1hmz | 01-Sep-88 |  |  |
| M_DEK | M | C | IDU | SC | 3DL1hmz | 15-Jun-93 |  |  |
| M_ITF | M | C | IDU | SC | 3DL1hmz | 01-Sep-88 |  |  |
| X_JXH | M | C | IDU | SC | 3DL1hmz | 01-Sep-88 |  |  |
| X_HXE | M | C | IDU | SC | 3DL1hmz | 15-Jan-93 |  |  |
| X_GLW | M | C | IDU | SC | 3DL1hmz | 15-Nov-96 |  |  |
| X_FTV | M | C | IDU | SC | 3DL1hmz | 15-Jan-95 |  |  |
| S_DGF | M | C | IDU | SC | 3DL1hmz | 15-Apr-00 |  |  |
| X_DCP | M | C | IDU | SC | 3DL1hmz | 15-Oct-02 |  |  |
| X_BAX | F | C | IDU | SC | 3DL1hmz | 01-Sep-88 |  |  |
| M_GXN | M | C | IDU | SC | 3DL1hmz | 01-Sep-88 |  |  |
| M_HKV | M | C | IDU | SC | 3DL1hmz | 01-Sep-88 |  |  |
| X_LTJ | F | C | IDU | SC | 3DL1hmz | 01-Sep-88 |  |  |
| H_AQS | F | C | IDU | SC | 3DL1hmz | 01-Sep-07 |  |  |
| H_AIO | M | C | IDU | SC | 3DL1hmz | 01-Sep-88 |  |  |
| X_HHF | M | C | IDU | SC | 3DL1hmz | 01-Sep-88 |  |  |
| DC_020 | F | C | HS | SC | 3DL1hmz | 30-Mar-85 |  |  |
| X_PBB | M | C | IDU | SC | 3DL1hmz | 15-Jul-98 |  |  |
| DC_022 | F | C | MSM | SC | 3DL1hmz | 01-Jun-88 |  |  |
| X_JVK | M | C | IDU | HESN | 3DL1/S1 | 15-Sep-96 | 20-Sep-02 | 2196 |
| S_JST | M | C | IDU | HESN | 3DL1/S1 | 30-Mar-00 | 24-Apr-06 | 2216 |
| H_ABA | M | C | IDU | HESN | 3DL1/S1 | 15-Jun-03 | 27-Oct-09 | 2326 |
| X_GID | M | C | IDU | HESN | 3DL1/S1 | 15-Jul-97 | 05-Nov-04 | 2670 |
| S_CXL | M | C | IDU | HESN | 3DL1/S1 | 15-Feb-02 | 16-Sep-09 | 2770 |
| X_EWW | F | C | IDU | HESN | 3DL1/S1 | 15-Apr-00 | 26-Jan-09 | 3208 |
| X_UFF | F | C | IDU | HESN | 3DL1/S1 | 15-Jul-91 | 06-Jul-00 | 3279 |
| X_CBZ | M | C | IDU | HESN | 3DL1/S1 | 01-Sep-88 | 17-Sep-02 | 5129 |
| X_MCI | M | C | IDU | HESN | 3DL1/S1 | 15-Oct-88 | 20-Feb-03 | 5241 |
| N_NLW | M | C | IDU | HESN | 3DL1/S1 | 01-Sep-88 | 25-Apr-03 | 5349 |
| X_FRR | M | C | IDU | HESN | 3DL1/S1 | 01-Sep-88 | 03-Mar-04 | 5662 |
| M_NVE | M | C | IDU | HESN | 3DL1/S1 | 01-Sep-88 | 28-Jun-05 | 6144 |
| X_CLA | M | C | IDU | HESN | 3DL1/S1 | 01-Sep-88 | 08-Sep-05 | 6216 |
| M_GBR | M | C | IDU | HESN | 3DL1/S1 | 01-Sep-88 | 05-Dec-05 | 6304 |
| M_NMP | M | C | IDU | HESN | 3DL1/S1 | 01-Sep-88 | 20-Feb-07 | 6746 |
| X_AKP | M | C | IDU | HESN | 3DL1/S1 | 01-Sep-88 | 13-Dec-07 | 7042 |
| H_ALT | M | C | IDU | HESN | 3DL1/S1 | 01-Sep-88 | 03-Feb-09 | 7460 |
| X_HCE | M | C | IDU | HESN | 3DL1/S1 | 01-Sep-88 | 23-Jul-09 | 7630 |
| M_EGH | F | C | IDU | HESN | 3DL1/S1 | 01-Sep-88 | 22-Sep-10 | 8056 |
| H_ABP | F | Native | IDU | HESN | 3DL1/S1 | 15-Jan-03 | 07-Jul-09 | 2365 |
| DC_007 | F | Native | HS | HESN | 3DL1/S1 | 01-Nov-99 | 01-Nov-01 | 731 |
| DC_008 | M | C | MSM | HESN | 3DL1/S1 | 01-Jan-96 | 01-Jan-98 | 731 |
| DC_009 | M | C | HS | HESN | 3DL1/S1 | 01-Nov-96 | 01-Mar-99 | 850 |
| DC_010 | M | C | MSM | HESN | 3DL1/S1 | 01-Jul-92 | 01-Jul-95 | 1095 |
| DC_011 | F | C | HS | HESN | 3DL1/S1 | 01-Jan-99 | 04-Apr-06 | 2650 |
| DC_012 | M | C | HS | HESN | 3DL1/S1 | 01-Jan-85 | 01-Jan-98 | 4748 |
| DC_013 | F | C | MSM | HESN | 3DL1/S1 | 01-Jan-96 | 01-Jan-98 | 731 |
| X_CVV | M | C | IDU | SC | 3DL1/S1 | 15-Jul-97 |  |  |
| X_NEN | M | C | IDU | SC | 3DL1/S1 | 15-Aug-97 |  |  |
| M_DNZ | M | C | IDU | SC | 3DL1/S1 | 15-Sep-95 |  |  |
| X_CAC | F | C | IDU | SC | 3DL1/S1 | 15-Jul-95 |  |  |
| H_ADL | M | C | IDU | SC | 3DL1/S1 | 15-Jun-03 |  |  |
| X_GHP | F | C | IDU | SC | 3DL1/S1 | 15-Jul-97 |  |  |
| X_CNT | M | C | IDU | SC | 3DL1/S1 | 15-Jun-99 |  |  |
| X_IWX | M | C | IDU | SC | 3DL1/S1 | 15-Dec-97 |  |  |
| M_NAT | M | C | IDU | SC | 3DL1/S1 | 01-Sep-88 |  |  |
| M_MPN | M | C | IDU | SC | 3DL1/S1 | 15-Jul-89 |  |  |
| X_EZV | M | C | IDU | SC | 3DL1/S1 | 15-Jul-91 |  |  |
| M_GNF | M | C | IDU | SC | 3DL1/S1 | 01-Sep-88 |  |  |
| M_AFJ | M | C | IDU | SC | 3DL1/S1 | 01-Sep-88 |  |  |
| M_CAE | M | C | IDU | SC | 3DL1/S1 | 01-Sep-88 |  |  |
| X_NVC | M | C | IDU | SC | 3DL1/S1 | 01-Sep-88 |  |  |
| M_IGL | M | C | IDU | SC | 3DL1/S1 | 01-Sep-88 |  |  |
| M_PAE | M | C | IDU | SC | 3DL1/S1 | 01-Sep-88 |  |  |
| X_IXN | M | C | IDU | SC | 3DL1/S1 | 15-Jan-90 |  |  |
| M_BRM | M | C | IDU | SC | 3DL1/S1 | 01-Sep-88 |  |  |
| M_KAW | M | C | IDU | SC | 3DL1/S1 | 15-Sep-90 |  |  |
| M_EWX | M | C | IDU | SC | 3DL1/S1 | 15-Jul-89 |  |  |
| X_GXKS | M | C | IDU | SC | 3DL1/S1 | 01-Sep-88 |  |  |
| M_EEG | M | C | IDU | SC | 3DL1/S1 | 01-Sep-88 |  |  |
| X_FRV | M | C | IDU | SC | 3DL1/S1 | 15-Jul-97 |  |  |
| M_EAK | M | C | IDU | SC | 3DL1/S1 | 01-Sep-88 |  |  |
| S_FIK | M | C | IDU | SC | 3DL1/S1 | 15-Jul-97 |  |  |
| 68_OPICP | M | C | IDU | SC | 3DL1/S1 | 08-Nov-06 |  |  |
| M_HZI | M | C | IDU | SC | 3DL1/S1 | 01-Sep-88 |  |  |
| DC_019 | M | C | MSM | SC | 3DL1/S1 | 19-Oct-91 |  |  |
| M_CBB | M | C | IDU | SC | 3DL1/S1 | 15-Jul-88 |  |  |
| X_PWH | M | C | IDU | SC | 3DL1/S1 | 15-Jun-99 |  |  |
| X_NNT | M | C | IDU | HESN | 3DS1hmz | 15-Nov-96 | 13-Nov-00 | 1459 |
| X_KJK | M | C | IDU | HESN | 3DS1hmz | 15-Mar-96 | 09-Nov-04 | 3161 |
| X_KGB | M | C | IDU | HESN | 3DS1hmz | 01-Sep-88 | 21-Mar-01 | 4584 |
| M_ABD | M | C | IDU | HESN | 3DS1hmz | 01-Sep-88 | 29-Aug-01 | 4745 |
| X_BBX | F | C | IDU | HESN | 3DS1hmz | 15-Jul-91 | 14-Sep-05 | 5175 |
| M_EZP | M | C | IDU | HESN | 3DS1hmz | 15-Jan-94 | 21-May-08 | 5240 |
| X_DDP | F | C | IDU | HESN | 3DS1hmz | 15-Oct-92 | 19-Feb-07 | 5240 |
| DC_014 | M | C | HS | HESN | 3DS1hmz | 01-Apr-95 | 01-Apr-96 | 366 |
| DC_015 | F | C | HS | HESN | 3DS1hmz | 01-Nov-95 | 01-Oct-97 | 700 |
| DC_016 | M | Native | MSM | HESN | 3DS1hmz | 01-Feb-92 | 01-Feb-95 | 1096 |
| DC_017 | M | C | MSM | HESN | 3DS1hmz | 01-Jul-90 | 25-Feb-09 | 6814 |
| DC_018 | F | C | HS | HESN | 3DS1hmz | 30-Mar-85 | 09-Feb-11 | 9447 |
| X_JWD | M | C | IDU | SC | 3DS1hmz | 15-Apr-93 |  |  |
| M_GGM | M | C | IDU | SC | 3DS1hmz | 01-Sep-88 |  |  |

**Table S1.** Study population characteristics. The identifying code, gender, ethnicity, risk group, serostatus category, *KIR3DL1/S1* genotype, the date of first needle sharing or sexual HIV exposure, the date of censoring for HIV exposed seronegative (HESN) subjects and the time, in days, from first HIV exposure to censoring for HESN is shown for each study subject.

^1^ M=male, F=female.

^2^ C=Caucasian, B-black, A= Asian, Native= Native American, other= not Caucasian, Black, Asian or Native American

^3^ IDU= Injection Drug User, MSM, men who have sex with men, HS= heterosexual.

^4^ HESN = HIV exposed seronegative, SC= Seroconverter.

^5^ 3DL1 hmz = *KIR3DL1* homozygote, 3DL1/3DS1 = *KIR3DL1/S1* heterozygotes, 3DS1 hmz = *KIR3DS1* homozygote.

^6^ Date at which subject first shared needles with others or September 1^st^, 1988, whichever is later. By September 1^st^, 1988 10% of IDU in Montreal were HIV positive.

^7^ Date of censoring was the date of last the clinic visit where an HESN reported sharing needles with an HIV infected partner in the last 6 months.

^8^  Interval in days between the first sharing and censoring date.
