# Supplementary material for: Time to Seroconversion in HIV-Exposed Subjects Carrying Protective versus Non Protective KIR3DS1/L1 and HLA-B Genotypes
Source: PLoS One. 2014 Oct 17;9(10):e110480. doi: 10.1371/journal.pone.0110480 (PMC4201542; doi:10.1371/journal.pone.0110480)
Supplement: Table S2 — Study population characteristics and KIR3DL1/S1 genotype for Seroconverters. (DOCX) [file pone.0110480.s003.docx]

**Table S2. Study population characteristics and *KIR3DL1/S1* genotype for Seroconverters**

| **ID** | **M/F^1^** | **Race^2^** | **Risk^3^** | **Serostatus^4^** | **3DL1/S1 Genotype^5^** |  | **First Sharing^6^** | **Seroconversion**  **Date^7^** | | **Time to SC^8^** |
| --- | --- | --- | --- | --- | --- | --- | --- | --- | --- | --- |
| X_BDYB | M | C | IDU | SC | 3DL1hmz |  | 13-Jun-01 | | 01-May-02 | 322 |
| M_MML | M | C | IDU | SC | 3DL1hmz |  | 15-Sep-95 | | 08-Apr-97 | 571 |
| X_HVK | M | C | IDU | SC | 3DL1hmz |  | 15-Jan-94 | | 06-Jun-97 | 1238 |
| X_RAI | M | C | IDU | SC | 3DL1hmz |  | 15-Jan-98 | | 14-May-02 | 1580 |
| X_EKL | M | C | IDU | SC | 3DL1hmz |  | 15-Jul-95 | | 15-Dec-99 | 1614 |
| M_MVB | M | C | IDU | SC | 3DL1hmz |  | 15-Jun-92 | | 06-Aug-97 | 1878 |
| M_DFH | M | C | IDU | SC | 3DL1hmz |  | 01-Sep-88 | | 27-Aug-96 | 2917 |
| X_FMH | M | C | IDU | SC | 3DL1hmz |  | 01-Sep-88 | | 15-Jul-97 | 3239 |
| X_KDX | M | C | IDU | SC | 3DL1hmz |  | 01-Sep-88 | | 15-Apr-97 | 3148 |
| X_MFM | M | C | IDU | SC | 3DL1hmz |  | 15-Jul-96 | | 26-Dec-02 | 2355 |
| M_FWB | F | C | IDU | SC | 3DL1hmz |  | 15-Dec-93 | | 06-Jun-02 | 3095 |
| M_PVN | M | C | IDU | SC | 3DL1hmz |  | 01-Sep-88 | | 04-Feb-98 | 3443 |
| M_KJE | M | C | IDU | SC | 3DL1hmz |  | 01-Sep-88 | | 31-Mar-99 | 3863 |
| M_NFC | M | C | IDU | SC | 3DL1hmz |  | 01-Sep-88 | | 08-Apr-99 | 3871 |
| M_KTC | M | C | IDU | SC | 3DL1hmz |  | 01-Sep-88 | | 18-Jun-99 | 3942 |
| X_LMA | M | C | IDU | SC | 3DL1hmz |  | 01-Sep-88 | | 19-Jul-00 | 4339 |
| X_PCV | M | C | IDU | SC | 3DL1hmz |  | 01-Sep-88 | | 01-Jul-99 | 3955 |
| M_LII | M | C | IDU | SC | 3DL1hmz |  | 15-Sep-93 | | 10-Mar-04 | 3829 |
| X_NVJ | M | C | IDU | SC | 3DL1hmz |  | 15-Jan-98 | | 03-Apr-07 | 3365 |
| X_GKT | M | C | IDU | SC | 3DL1hmz |  | 01-Sep-88 | | 15-Jun-00 | 4305 |
| X_ZMYK | M | C | IDU | SC | 3DL1hmz |  | 15-Jul-96 | | 21-Mar-06 | 3536 |
| M_LFW | M | C | IDU | SC | 3DL1hmz |  | 01-Sep-88 | | 04-Jun-01 | 4659 |
| X_IRM | F | C | IDU | SC | 3DL1hmz |  | 01-Sep-88 | | 01-Jul-01 | 4686 |
| M_DEK | M | C | IDU | SC | 3DL1hmz |  | 15-Jun-93 | | 12-Jan-04 | 3863 |
| M_ITF | M | C | IDU | SC | 3DL1hmz |  | 01-Sep-88 | | 05-Feb-04 | 5635 |
| X_JXH | M | C | IDU | SC | 3DL1hmz |  | 01-Sep-88 | | 01-Aug-03 | 5447 |
| X_HXE | M | C | IDU | SC | 3DL1hmz |  | 15-Jan-93 | | 18-Mar-08 | 5541 |
| X_GLW | M | C | IDU | SC | 3DL1hmz |  | 15-Nov-96 | | 21-Dec-96 | 36 |
| X_PWH | M | C | IDU | SC | 3DL1hmz |  | 15-Jun-99 | | 27-Apr-00 | 317 |
| X_FTV | M | C | IDU | SC | 3DL1hmz |  | 15-Jan-95 | | 14-Oct-97 | 1003.5 |
| S_DGF | M | C | IDU | SC | 3DL1hmz |  | 15-Apr-00 | | 28-Oct-03 | 1291 |
| X_DCP | M | C | IDU | SC | 3DL1hmz |  | 15-Oct-02 | | 05-Mar-07 | 1602 |
| X_BAX | F | C | IDU | SC | 3DL1hmz |  | 01-Sep-88 | | 31-Oct-98 | 3712 |
| M_GXN | M | C | IDU | SC | 3DL1hmz |  | 01-Sep-88 | | 20-Jan-99 | 3793.5 |
| M_HKV | M | C | IDU | SC | 3DL1hmz |  | 01-Sep-88 | | 12-Oct-02 | 5154 |
| X_LTJ | F | C | IDU | SC | 3DL1hmz |  | 01-Sep-88 | | 21-Aug-96 | 2911 |
| H_AQS | F | C | IDU | SC | 3DL1hmz |  | 01-Sep-07 | | 19-Dec-07 | 109 |
| H_AIO | M | C | IDU | SC | 3DL1hmz |  | 01-Sep-88 | | 27-Jun-06 | 6508 |
| X_HHF | M | C | IDU | SC | 3DL1hmz |  | 01-Sep-88 | | 09-Jul-98 | 3598 |
| DC_020 | F | C | HS | SC | 3DL1hmz |  | 30-Mar-85 | | 30-Mar-90 | 1826 |
| X_PBB | M | C | IDU | SC | 3DL1hmz |  | 15-Jul-98 | | 06-Nov-02 | 1575 |
| DC_022 | F | C | MSM | SC | 3DL1hmz |  | 01-Jun-88 | | 17-Nov-97 | 3456 |
| X_CVV | M | C | IDU | SC | 3DL1/S1 |  | 15-Jul-97 | | 03-Feb-98 | 203 |
| X_NEN | M | C | IDU | SC | 3DL1/S1 |  | 15-Aug-97 | | 24-Mar-98 | 221 |
| M_DNZ | M | C | IDU | SC | 3DL1/S1 |  | 15-Sep-95 | | 26-Jun-97 | 650 |
| X_CAC | F | C | IDU | SC | 3DL1/S1 |  | 15-Jul-95 | | 22-Aug-97 | 769 |
| H_ADL | M | C | IDU | SC | 3DL1/S1 |  | 15-Jun-03 | | 03-Nov-06 | 1237 |
| X_GHP | F | C | IDU | SC | 3DL1/S1 |  | 15-Jul-97 | | 25-Oct-00 | 1198 |
| X_CNT | M | C | IDU | SC | 3DL1/S1 |  | 15-Jun-99 | | 30-May-02 | 1080 |
| X_IWX | M | C | IDU | SC | 3DL1/S1 |  | 15-Dec-97 | | 01-Oct-02 | 1751 |
| M_NAT | M | C | IDU | SC | 3DL1/S1 |  | 01-Sep-88 | | 21-Mar-96 | 2758 |
| M_MPN | M | C | IDU | SC | 3DL1/S1 |  | 15-Jul-89 | | 15-Jun-96 | 2527 |
| X_EZV | M | C | IDU | SC | 3DL1/S1 |  | 15-Jul-91 | | 15-Nov-96 | 1950 |
| M_GNF | M | C | IDU | SC | 3DL1/S1 |  | 01-Sep-88 | | 12-Nov-96 | 2994 |
| M_AFJ | M | C | IDU | SC | 3DL1/S1 |  | 01-Sep-88 | | 09-Jun-97 | 3203 |
| M_CAE | M | C | IDU | SC | 3DL1/S1 |  | 01-Sep-88 | | 22-Sep-97 | 3308 |
| X_NVC | M | C | IDU | SC | 3DL1/S1 |  | 01-Sep-88 | | 07-Jul-97 | 3231 |
| M_IGL | M | C | IDU | SC | 3DL1/S1 |  | 01-Sep-88 | | 15-Jul-97 | 3239 |
| M_PAE | M | C | IDU | SC | 3DL1/S1 |  | 01-Sep-88 | | 10-Feb-98 | 3449 |
| X_IXN | M | C | IDU | SC | 3DL1/S1 |  | 15-Jan-90 | | 25-Feb-99 | 3328 |
| M_BRM | M | C | IDU | SC | 3DL1/S1 |  | 01-Sep-88 | | 15-Oct-99 | 4061 |
| M_KAW | M | C | IDU | SC | 3DL1/S1 |  | 15-Sep-90 | | 01-Nov-00 | 3700 |
| M_EWX | M | C | IDU | SC | 3DL1/S1 |  | 15-Jul-89 | | 24-Apr-01 | 4301 |
| X_GXKS | M | C | IDU | SC | 3DL1/S1 |  | 01-Sep-88 | | 15-Sep-01 | 4762 |
| M_EEG | M | C | IDU | SC | 3DL1/S1 |  | 01-Sep-88 | | 01-Jul-03 | 5416 |
| X_FRV | M | C | IDU | SC | 3DL1/S1 |  | 15-Jul-97 | | 09-Jun-00 | 1060 |
| M_EAK | M | C | IDU | SC | 3DL1/S1 |  | 01-Sep-88 | | 22-Jul-00 | 4342 |
| S_FIK | M | C | IDU | SC | 3DL1/S1 |  | 15-Jul-97 | | 06-Aug-97 | 22 |
| 68_OPICP | M | C | IDU | SC | 3DL1/S1 |  | 08-Nov-06 | | 09-May-07 | 182 |
| M_HZI | M | C | IDU | SC | 3DL1/S1 |  | 01-Sep-88 | | 23-Oct-93 | 1878 |
| DC_019 | M | C | MSM | SC | 3DL1/S1 |  | 19-Oct-91 | | 28-Nov-00 | 3328 |
| M_CBB | M | C | IDU | SC | 3DL1/S1 |  | 15-Jul-88 | | 15-Jan-97 | 3106 |
| X_PWH | M | C | IDU | SC | 3DL1/S1 |  | 15-Jun-99 | | 27-Apr-00 | 317 |
| X_JWD | M | C | IDU | SC | 3DS1hmz |  | 15-Apr-93 | | 01-Mar-00 | 2512 |
| M_GGM | M | C | IDU | SC | 3DS1hmz |  | 01-Sep-88 | | 01-Jul-98 | 3590 |

**Table S2.** Study population characteristics for seroconvertors. The identifying code, gender, ethnicity, risk group, serostatus category, *KIR3DL1/S1* genotype, the date of first needle sharing or sexual HIV exposure, the date of seroconversion and the time, in days, from first HIV exposure to seroconversion for seroconvertors is shown for each study subject.

^1^ M=male, F=female.

^2^ C=Caucasian

^3^ IDU= Injection Drug User, MSM, men who have sex with men, HS= heterosexual.

^4^ SC= Seroconverter.

^5^ 3DL1 hmz = *KIR3DL1* homozygote, 3DL1/3DS1 = *KIR3DL1/S1* heterozygotes, 3DS1 hmz = *KIR3DS1* homozygote.

^6^ Date at which subject first shared needles with others or September 1^st^, 1988, whichever is later. By September 1^st^, 1988 10% of IDU in Montreal were HIV positive.

^7^ Date of seroconversion. See methods section for details

^8^  Interval in days between the first sharing and seroconversion date.
