# Supplementary material for: Time to Seroconversion in HIV-Exposed Subjects Carrying Protective versus Non Protective KIR3DS1/L1 and HLA-B Genotypes
Source: PLoS One. 2014 Oct 17;9(10):e110480. doi: 10.1371/journal.pone.0110480 (PMC4201542; doi:10.1371/journal.pone.0110480)
Supplement: Table S3 — Genotype Group of Subjects with Copy Number Variation for KIR3DL1/S1. (DOCX) [file pone.0110480.s004.docx]

**Table S3: Genotype Group of Subjects with Copy Number Variation for *KIR3DL1/S1***

| Subject | Serostatus | Genotype | 3DL1 Copies | 3DS1 Copies | Time to Event (Days) |
| --- | --- | --- | --- | --- | --- |
|  |  |  |  |  |  |
|  |  |  |  |  |  |
| M_JZC | Negative | *3DL1* Hmz | 1 | 0 | 5278 |
| X_CFV | Negative | *3DL1* Hmz | 1 | 0 | 1049 |
| M_KJE | Positive | *3DL1* Hmz | 1 | 0 | 3863 |
| H_ALT | Negative | *3DL1/S1* Het | 2 | 1 | 7460 |
| X_MCI | Negative | *3DL1/S1* Het | 2 | 1 | 5241 |
| H_HZI | Positive | *3DL1/S1* Het | 1 | 2 | 1878 |
| M_MPN | Positive | *3DL1/S1* Het | 1 | 2 | 2527 |
| X_EWW | Negative | *3DL1/S1* Het | 1 | 2 | 3208 |
| M_PAE | Positive | *3DL1/S1* Het | 3 | 1 | 3449 |
| M_ABD | Negative | *3DS1* Hmz | 0 | 1 | 4745 |
|  |  |  |  |  |  |
|  |  |  |  |  |  |

**Table S3.** Ten study subjects had copy number variation at the *KIR3DS1/L1* locus. Their HIV serostatus (HIV negative or positive), *KIR3DL1/S1* genotype, number of *KIR3DL1 (3DL1)* and *KIR3DS1 (3DS1)* copies they carried and time, in days, from first exposure to event (censoring or seroconversion) is shown. *3DL1* hmz = *KIR3DL1* homozygote, *3DL1/S1* het = *KIR3DL1/S1* heterozygote, *3DS1* hmz = *KIR3DS1* homozygote.
