# Supplementary material for: Time to Seroconversion in HIV-Exposed Subjects Carrying Protective versus Non Protective KIR3DS1/L1 and HLA-B Genotypes
Source: PLoS One. 2014 Oct 17;9(10):e110480. doi: 10.1371/journal.pone.0110480 (PMC4201542; doi:10.1371/journal.pone.0110480)
Supplement: Table S4 — Study population HLA types and KIR/HLA genotype categoriess used in analyses. (DOCX) [file pone.0110480.s005.docx]

**Table S4. Study population HLA types and KIR/HLA genotype categoriess used in analyses**.

| **ID** | **Serostatus^1^** | ***3DL1/S1* Genotype^2^** | | ***HLA-A*** | ***HLA-A*** | | ***HLA-B*** | ***HLA-B*** | ****h/*y+B*57*^3^** | **Not**  ****h/*y+B*57*^4^** | ***3DS1 +***  ***Bw4*80I*^5^** | ***Bw6*^6^** |
| --- | --- | --- | --- | --- | --- | --- | --- | --- | --- | --- | --- | --- |
| H_ANN | HESN | 3DL1hmz | *01:01 | | | *02:01 | *07:02 | *37:01 |  | ● |  |  |
| X_BDE | HESN | 3DL1hmz | *26:01 | | | *68:01 | *07:02 | *38:01 |  | ● |  |  |
| M_LRB | HESN | 3DL1hmz | *24:02 | | | *24:02 | *39:01 | *49:01 |  | ● |  |  |
| M_HAS | HESN | 3DL1hmz | *02:01 | | | *02:01 | *44:02 | *44:02 |  | ● |  |  |
| X_ADT | HESN | 3DL1hmz | *01:01 | | | *03:01 | *40:01 | *44:24 |  | ● |  |  |
| S_RCK | HESN | 3DL1hmz | *11:01 | | | *24:02 | *27:05 | *53:01 |  | ● |  |  |
| X_ADX | HESN | 3DL1hmz | *01:01 | | | *26:01 | *08:01 | *08:01 |  | ● |  | ● |
| M_MMZ | HESN | 3DL1hmz | *02:01 | | | *24:02 | *51:01 | *51:01 |  | ● |  |  |
| M_EAR | HESN | 3DL1hmz | *11:01 | | | *29:01 | *39:01 | *44:03 |  | ● |  |  |
| X_JCA | HESN | 3DL1hmz | *01:01 | | | *01:01 | *07:02 | *39:01 |  | ● |  | ● |
| X_FHV | HESN | 3DL1hmz | *02:01 | | | *03:01 | *07:02 | *40:01 |  | ● |  | ● |
| M_JEF | HESN | 3DL1hmz | *24:02 | | | *26:01 | *35:01 | *51:01 |  | ● |  |  |
| X_MIW | HESN | 3DL1hmz | *02:01 | | | *23:01 | *37:01 | *49:01 |  | ● |  |  |
| X_EMX | HESN | 3DL1hmz | *01:01 | | | *02:01 | *38:01 | *57:01 | ● |  |  |  |
| X_ITL | HESN | 3DL1hmz | *29:01 | | | *68:-01 | *44:03 | *57:01 | ● |  |  |  |
| M_LHE | HESN | 3DL1hmz | *02:01 | | | *24:02 | *07:02 | *58:01 |  | ● |  |  |
| X_DLH | HESN | 3DL1hmz | *29:01 | | | *31:01 | *40:01 | *44:03 |  | ● |  |  |
| M_JZC | HESN | 3DL1hmz | *03:01 | | | *32:01 | *07:02 | *44:03 |  | ● |  |  |
| M_LZC | HESN | 3DL1hmz | *02:01 | | | *29:01 | *39:01 | *50:01 |  | ● |  | ● |
| X_BWC | HESN | 3DL1hmz | *31:01 | | | *68:01 | *27:08 | *51:01 |  | ● |  |  |
| X_DFN | HESN | 3DL1hmz | *68:01 | | | *68:01 | *15:01 | *44:02 |  | ● |  |  |
| X_LYZW | HESN | 3DL1hmz | *01:01 | | | *03:01 | *07:02 | *27:05 |  | ● |  |  |
| M_AIB | HESN | 3DL1hmz | *02:01 | | | *02:01 | *07:02 | *14:01 |  | ● |  | ● |
| M_BCR | HESN | 3DL1hmz | *03:01 | | | *32:01 | *07:02 | *27:05 |  | ● |  |  |
| X_GCA | HESN | 3DL1hmz | *02:22 | | | *03:01 | *44:02 | *57:01 | ● |  |  |  |
| M_GEF | HESN | 3DL1hmz | *02:01 | | | *02:01 | *18:01 | *40:01 |  | ● |  | ● |
| X_FLI | HESN | 3DL1hmz | *11:01 | | | *68:01 | *15:10 | *35:01 |  | ● |  | ● |
| M_NSZ | HESN | 3DL1hmz | *01:01 | | | *24:03 | *07:02 | *07:02 |  | ● |  |  |
| X_AGI | HESN | 3DL1hmz | *03:01 | | | *11:01 | *07:02 | *35:01 |  | ● |  | ● |
| X_CDC | HESN | 3DL1hmz | *01:01 | | | *29:01 | *44:03 | *18:01 |  | ● |  |  |
| M_BMF | HESN | 3DL1hmz | *01:01 | | | *02:01 | *15:01 | *08:01 |  | ● |  | ● |
| X_NSX | HESN | 3DL1hmz | *01:01 | | | *11:01 | *51:01 | *55:01 |  | ● |  |  |
| X_EHD | HESN | 3DL1hmz | *29:01 | | | *32:01 | *14:02 | *44:03 |  | ● |  |  |
| M_LEG | HESN | 3DL1hmz | *02:01 | | | *03:01 | *07:02 | *15:01 |  | ● |  | ● |
| X_IBL | HESN | 3DL1hmz | *02:01 | | | *02:01 | *55:01 | *55:01 |  | ● |  | ● |
| M_CGX | HESN | 3DL1hmz | *02:01 | | | *32:01 | *18:01 | *50:01 |  | ● |  |  |
| X_YDKC | HESN | 3DL1hmz | *01:01 | | | *02:01 | *08:01 | *08:01 |  | ● |  | ● |
| M_EHF | HESN | 3DL1hmz | *24:02 | | | *25:01 | *37:01 | *57:01 | ● |  |  |  |
| X_RHG | HESN | 3DL1hmz | *02:01 | | | *25:01 | *18:01 | *55:01 |  | ● |  |  |
| H_ALA | HESN | 3DL1hmz | *02:05 | | | *03:01 | *37:01 | B*41:01 |  | ● |  |  |
| X_LTG | HESN | 3DL1hmz | *23:01 | | | *68:01 | *37:01 | *44:03 |  | ● |  |  |
| M_AOD | HESN | 3DL1hmz | *02:01 | | | *02:01 | *57:01 | *57:01 | ● |  |  |  |
| DC_001 | HESN | 3DL1hmz | *11:01 | | | *24:02 | *40:01 | *51:01 |  | ● |  |  |
| DC_002 | HESN | 3DL1hmz | *03:01 | | | *31:02 | *35:01 | *44:03 |  | ● |  |  |
| DC_003 | HESN | 3DL1hmz | *24:02 | | | *24:02 | *44:02 | *27:02 |  | ● |  |  |
| DC_004 | HESN | 3DL1hmz | *01:01 | | | *02:01 | *08:01 | *49:01 |  | ● |  |  |
| DC_005 | HESN | 3DL1hmz | *02:01 | | | *32:01 | *07:02 | *08:01 |  | ● |  |  |
| DC_006 | HESN | 3DL1hmz | *01:01 | | | *02:01 | *38:01 | *51:01 |  | ● |  |  |
| DC_021 | HESN | 3DL1hmz | *01:01 | | | *02:01 | *07:02 | *27:05 |  | ● |  |  |
| M_LDN | HESN | 3DL1hmz | *01:01 | | | *26:01 | *38:01 | *57:01 | ● |  |  |  |
| X_CFV | HESN | 3DL1hmz | *29:01 | | | *36:01 | *07:02 | *53:01 |  | ● |  |  |
| X_DHZ | HESN | 3DL1hmz | *02:01 | | | *24:02 | *15:01 | *15:01 |  | ● |  |  |
| X_HPJ | HESN | 3DL1hmz | *02:01 | | | *03:01 | *35:01 | *51:01 |  | ● |  |  |
| X_IIM | HESN | 3DL1hmz | *02:01 | | | *24:02 | *14:02 | *27:05 |  | ● |  |  |
| X_BDYB | SC | 3DL1hmz | *32:01 | | | *34:01 | *08:01 | *15:01 |  | ● |  |  |
| M_MML | SC | 3DL1hmz | *11:01 | | | *11:01 | *35:01 | *44:02 |  | ● |  |  |
| X_HVK | SC | 3DL1hmz | *02:01 | | | *03:01 | *35:01 | *51:01 |  | ● |  |  |
| X_RAI | SC | 3DL1hmz | *03:01 | | | *29:01 | *40:01 | *44:03 |  | ● |  |  |
| X_EKL | SC | 3DL1hmz | *01:01 | | | *29:02 | *08:01 | *44:03 |  | ● |  |  |
| M_MVB | SC | 3DL1hmz | *02:01 | | | *02:01 | *44:02 | *44:02 |  | ● |  |  |
| M_DFH | SC | 3DL1hmz | *01:01 | | | *68:01 | *57:01 | *27:05 | ● |  |  |  |
| X_FMH | SC | 3DL1hmz | *01:01 | | | *02:01 | *07:02 | *37:01 |  | ● |  |  |
| X_KDX | SC | 3DL1hmz | *24:02 | | | *68:01 | *35:05 | *44:02 |  | ● |  |  |
| X_MFM | SC | 3DL1hmz | *02:01 | | | *29:01 | *27:05 | *45:01 |  | ● |  |  |
| M_FWB | SC | 3DL1hmz | *02:01 | | | *03:01 | *44:02 | *49:01 |  | ● |  |  |
| M_PVN | SC | 3DL1hmz | *11:01 | | | *31:01 | *15:08 | *39:01 |  | ● |  | ● |
| M_KJE | SC | 3DL1hmz | *02:01 | | | *26:01 | *27:05 | *35:01 |  | ● |  |  |
| M_NFC | SC | 3DL1hmz | *02:01 | | | *03:01 | *07:02 | *40:02 |  | ● |  | ● |
| M_KTC | SC | 3DL1hmz | *02:01 | | | *11:01 | *51:01 | *51:01 |  | ● |  |  |
| X_LMA | SC | 3DL1hmz | *02:01 | | | *03:01 | *15:01 | *49:01 |  | ● |  |  |
| X_PCV | SC | 3DL1hmz | *03:01 | | | *66:01 | *18:01 | *41:01 |  | ● |  | ● |
| M_LII | SC | 3DL1hmz | *02:01 | | | *02:01 | *08:01 | *44:02 |  | ● |  |  |
| X_NVJ | SC | 3DL1hmz | *23:01 | | | *25:01 | *08:01 | *44:03 |  | ● |  |  |
| X_GKT | SC | 3DL1hmz | *31:01 | | | *33:01 | *14:02 | *40:01 |  | ● |  | ● |
| X_ZMYK | SC | 3DL1hmz | *01:01 | | | *01:01 | *08:01 | *44:02 |  | ● |  |  |
| M_LFW | SC | 3DL1hmz | *02:01 | | | *03:01 | *15:01 | *14:02 |  | ● |  | ● |
| X_IRM | SC | 3DL1hmz | *02:01 | | | *30:01 | *13:01 | *35:02 |  | ● |  |  |
| M_DEK | SC | 3DL1hmz | *02:01 | | | *30:01 | *18:01 | *44:02 |  | ● |  |  |
| M_ITF | SC | 3DL1hmz | *03:01 | | | *03:01 | *07:02 | *14:02 |  | ● |  | ● |
| X_JXH | SC | 3DL1hmz | *01:01 | | | *31:01 | *35:01 | *40:01 |  | ● |  | ● |
| X_HXE | SC | 3DL1hmz | *02:01 | | | *31:01 | *27:05 | *40:01 |  | ● |  |  |
| X_GLW | SC | 3DL1hmz | *30:01 | | | *68:01 | *40:01 | *44:02 |  | ● |  |  |
| X_FTV | SC | 3DL1hmz | *01:01 | | | *32:01 | *07:02 | *51:01 |  | ● |  |  |
| S_DGF | SC | 3DL1hmz | *02:01 | | | *02:01 | *44:02 | *44:02 |  | ● |  |  |
| X_DCP | SC | 3DL1hmz | *02:01 | | | *24:02 | *44:02 | *51:01 |  | ● |  |  |
| X_BAX | SC | 3DL1hmz | *02:01 | | | *03:01 | *07:02 | *40:02 |  | ● |  | ● |
| M_GXN | SC | 3DL1hmz | *24:02 | | | *31:01 | *18:01 | *27:05 |  | ● |  |  |
| M_HKV | SC | 3DL1hmz | *03:01 | | | *23:01 | *15:01 | *07:02 |  | ● |  |  |
| X_LTJ | SC | 3DL1hmz | *01:01 | | | *29:01 | *35:01 | *40:04 |  | ● |  | ● |
| H_AQS | SC | 3DL1hmz | *03:01 | | | *29:01 | *35:02 | *44:03 |  | ● |  |  |
| H_AIO | SC | 3DL1hmz | *23:01 | | | *29:01 | *15:01 | *56:02 |  | ● |  |  |
| X_HHF | SC | 3DL1hmz | *03:01 | | | *30:02 | *08:01 | *49:01 |  | ● |  |  |
| DC_020 | SC | 3DL1hmz | *01:01 | | | *03:01 | *49:01 | *57:01 |  | ● |  |  |
| X_PBB | SC | 3DL1hmz | *23:01 | | | *34:02 | *35:01 | *58:02 |  | ● |  |  |
| DC_022 | SC | 3DL1hmz | *11:01 | | | *26:01 | *27:05 | *51:01 |  | ● |  |  |
| X_JVK | HESN | 3DL1/S1 | *01:01 | | | *25:01 | *08:01 | *08 or *55 |  | ● | ■ |  |
| S_JST | HESN | 3DL1/S1 | *02:01 | | | *02:01 | *44:02 | *44:03 |  | ● |  |  |
| H_ABA | HESN | 3DL1/S1 | *02:01 | | | *03:01 | *07:02 | *35:03 |  | ● |  | ● |
| X_GID | HESN | 3DL1/S1 | *01:01 | | | *24:02 | *35:01 | *52:01 |  | ● | ● |  |
| S_CXL | HESN | 3DL1/S1 | *31:02 | | | *68:01 | *40:01 | *40:01 |  | ● |  | ● |
| X_EWW | HESN | 3DL1/S1 | *02:01 | | | *03:01 | *07:02 | *07:02 |  | ● |  | ● |
| X_UFF | HESN | 3DL1/S1 | *26:01 | | | *68:01 | *07:02 | *08:01 |  | ● |  | ● |
| X_CBZ | HESN | 3DL1/S1 | *03:01 | | | *23:01 | *07:02 | *45:01 |  | ● | ■ |  |
| X_MCI | HESN | 3DL1/S1 | *02:01 | | | *02:01 | *27:05 | *27:05 |  | ● |  |  |
| N_NLW | HESN | 3DL1/S1 | *02:01 | | | *02:01 | *40:01 | *44:02 |  | ● |  |  |
| X_FRR | HESN | 3DL1/S1 | *02:01 | | | *26:01 | *38:01 | *44:02 |  | ● | ● |  |
| M_NVE | HESN | 3DL1/S1 | *03:01 | | | *68:01 | *14:02 | *15:01 |  | ● |  | ● |
| X_CLA | HESN | 3DL1/S1 | *29:01 | | | *32:01 | *15:01 | *44:03 |  | ● | ■ |  |
| M_GBR | HESN | 3DL1/S1 | *02:01 | | | *02:01 | *44:02 | *49:01 |  | ● | ● |  |
| M_NMP | HESN | 3DL1/S1 | *25:01 | | | *68:01 | *18:01 | *44:02 |  | ● | ■ |  |
| X_AKP | HESN | 3DL1/S1 | *02:01 | | | *02:01 | *44:02 | *15:01 |  | ● |  |  |
| H_ALT | HESN | 3DL1/S1 | *02:01 | | | *02:01 | *15:01 | *51:01 |  | ● | ● |  |
| X_HCE | HESN | 3DL1/S1 | *03:01 | | | *33:01 | *53:01 | *14:02 |  | ● | ● |  |
| M_EGH | HESN | 3DL1/S1 | *02:01 | | | *02:01 | *07:02 | *39:01 |  | ● |  | ● |
| H_ABP | HESN | 3DL1/S1 | *01:01 | | | *68:02 | *08:01 | *44:02 |  | ● |  |  |
| DC_007 | HESN | 3DL1/S1 | *03:01 | | | *24:02 | *07:02 | *40:02 |  | ● | ■ |  |
| DC_008 | HESN | 3DL1/S1 | *03:01 | | | *31:01 | *40:01 | *40:01 |  | ● |  | ● |
| DC_009 | HESN | 3DL1/S1 | *02:01 | | | *24:02 | *15:01 | *44:02 |  | ● | ■ |  |
| DC_010 | HESN | 3DL1/S1 | *02:01 | | | *03:01 | *44:03 | *08:01 |  | ● |  |  |
| DC_011 | HESN | 3DL1/S1 | *02:01 | | | *03:01 | *15:01 | *07:02 |  | ● |  | ● |
| DC_012 | HESN | 3DL1/S1 | *32:01 | | | *68:01 | *35:03 | *35:03 |  | ● | ■ |  |
| DC_013 | HESN | 3DL1/S1 | *01:01 | | | *02:01 | *08:01 | *15:01 |  | ● |  | ● |
| X_CVV | SC | 3DL1/S1 | *03:01 | | | *24:02 | *07:02 | *18:01 |  | ● | ■ |  |
| X_NEN | SC | 3DL1/S1 | *03:01 | | | *25:01 | *18:01 | *44:02 |  | ● | ■ |  |
| M_DNZ | SC | 3DL1/S1 | *23:01 | | | *29:01 | *44:03 | *49:01 |  | ● | ● |  |
| X_CAC | SC | 3DL1/S1 | *01:01 | | | *03:01 | *08:01 | *14:02 |  | ● |  | ● |
| H_ADL | SC | 3DL1/S1 | *02:01 | | | *02:01 | *18:01 | *27:05 |  | ● |  |  |
| X_GHP | SC | 3DL1/S1 | *02:01 | | | *02:01 | *27:05 | *49:01 |  | ● | ● |  |
| X_CNT | SC | 3DL1/S1 | *29:01 | | | *03:01 | *07:02 | *44:03 |  | ● |  |  |
| X_IWX | SC | 3DL1/S1 | *24:02 | | | *29:01 | *44:03 | *49:01 |  | ● | ● |  |
| M_NAT | SC | 3DL1/S1 | *02:01 | | | *24:02 | *27:05 | *40:01 |  | ● | ■ |  |
| M_MPN | SC | 3DL1/S1 | *02:01 | | | *34 | *44:02 | *18:01 |  | ● |  |  |
| X_EZV | SC | 3DL1/S1 | *01:01 | | | *02:01 | *35:01 | *51:01 |  | ● | ● |  |
| M_GNF | SC | 3DL1/S1 | *01:01 | | | *33:01 | *44:03 | *14:02 |  | ● |  |  |
| M_AFJ | SC | 3DL1/S1 | *02:01 | | | *02:01 | *40:01 | *44:02 |  | ● |  |  |
| M_CAE | SC | 3DL1/S1 | *02:01 | | | *32:01 | *44:03 | *55:01 |  | ● | ■ |  |
| X_NVC | SC | 3DL1/S1 | *02:01 | | | *29 or *26 | *14 | *35 |  | ● |  | ● |
| M_IGL | SC | 3DL1/S1 | *02:01 | | | *03:01 | *27:05 | *40:02 |  | ● |  |  |
| M_PAE | SC | 3DL1/S1 | *02:01 | | | *03:01 | *40:01 | *49:01 |  | ● | ● |  |
| X_IXN | SC | 3DL1/S1 | *01:01 | | | *31:02 | *07:02 | *57:01 |  | ● | ● |  |
| M_BRM | SC | 3DL1/S1 | *02:01 | | | *26:01 | *49:01 | *51:01 |  | ● | ● |  |
| M_KAW | SC | 3DL1/S1 | *23:01 | | | *24:02 | *18:01 | *49:01 |  | ● | ● |  |
| M_EWX | SC | 3DL1/S1 | *02:01 | | | *02:01 | *18:01 | *39:01 |  | ● |  | ● |
| X_GXKS | SC | 3DL1/S1 | *02:01 | | | *24:02 | *18:01 | *40:02 |  | ● | ■ |  |
| M_EEG | SC | 3DL1/S1 | *01:01 | | | *26:01 | *08:01 | *14:01 |  | ● |  | ● |
| X_FRV | SC | 3DL1/S1 | *11:01 | | | *24:02 | *35:01 | *35:01 |  | ● | ■ |  |
| M_EAK | SC | 3DL1/S1 | *03:01 | | | *31:01 | *07:02 | *51:01 |  | ● | ● |  |
| S_FIK | SC | 3DL1/S1 | *02:01 | | | *68:01 | *14:02 | *44:02 |  | ● |  |  |
| 68_OPICP | SC | 3DL1/S1 | *11:01 | | | *69:01 | *27:05 | *35:01 |  | ● |  |  |
| M_HZI | SC | 3DL1/S1 | *02:01 | | | *03:01 | *08:01 | *44:02 |  | ● |  |  |
| DC_019 | SC | 3DL1/S1 | *01:01 | | | *03:01 | *49:01 | *57:01 |  | ● | ● |  |
| M_CBB | SC | 3DL1/S1 | *02:01 | | | *26:01 | *35:01 | *35:01 |  | ● |  |  |
| X_PWH | SC | 3DL1hmz | *24:02 | | | *26:01 | *15:01 | *27:05 |  | ● |  |  |
| X_NNT | HESN | 3DS1hmz | *02:01 | | | *03:01 | *07:02 | *56:01 |  |  |  |  |
| X_KJK | HESN | 3DS1hmz | *03:01 | | | *30:01 | *27:04 | *35:01 |  |  |  |  |
| X_KGB | HESN | 3DS1hmz | *01:01 | | | *01:01 | *08:01 | *38:01 |  |  |  |  |
| M_ABD | HESN | 3DS1hmz | *24:02 | | | *26:01 | *35:01 | *15:01 |  |  |  |  |
| X_BBX | HESN | 3DS1hmz | *01:01 | | | *01:01 | *08:01 | *57:01 |  |  |  |  |
| M_EZP | HESN | 3DS1hmz | *02:01 | | | *23:01 | *07:02 | *35:03 |  |  |  |  |
| X_DDP | HESN | 3DS1hmz | *02:01 | | | *23:01 | *40:02 | *07:02 |  |  |  |  |
| DC_014 | HESN | 3DS1hmz | *03:01 | | | *24:02 | *15:01 | *35:01 |  |  |  |  |
| DC_015 | HESN | 3DS1hmz | *02:01 | | | *31:01 | *14:01 | *15:01 |  |  |  |  |
| DC_016 | HESN | 3DS1hmz | *02:01 | | | *24:02 | *07:02 | *35:01 |  |  |  |  |
| DC_017 | HESN | 3DS1hmz | *31:01 | | | *68:02 | *38:01 | *51:01 |  |  |  |  |
| DC_018 | HESN | 3DS1hmz | *01:01 | | | *02:01 | *08:01 | *27:05 |  |  |  |  |
| X_JWD | SC | 3DS1hmz | *03:01 | | | *68:01 | *07:02 | *27:05 |  |  |  |  |
| M_GGM | SC | 3DS1hmz | *11:01 | | | *32:01 | *44:02 | *51:01 |  |  |  |  |

**Table S4.** Shown in this table for all study participants are their serostatus category, *KIR3DL1/S1* genotype, the *HLA-A* and *HLA-B* alleles they carried, which carried the **h/*y+B*57* genotype*, which* did not carry the **h/*y+B*57 genotype,* which carried *KIR3DS1+*80I* genotypes and which were *Bw6* homozygotes with no HLA-A Bw4 alleles*.*

^1^ HESN= HIV exposed seronegative, SC= Seroconverter.

^2^ 3DL1 hmz = *KIR3DL1* homozygote, 3DL1/3DS1 = *KIR3DL1/S1* heterozygotes, 3DS1 hmz = *KIR3DS1* homozygote.

^3^ ● indicate the subjects who carry the **h/*y+B*57* KIR/HLA genotype combination.

^4^ ● indicate the subjects who carry the *KIR3DL1* homozygous or *KIR3DL1/S1* heterozygous *KIR/HLA* genotype combinations that are not **h/*y+B*57*.

^5^  ● indicate the subjects who carry 1 copy of *KIR3DS1* and a *Bw4*80I* allele at the *HLA B* locus; ■ indicate the subjects who carry 1 copy of *KIR3DS1* and a *Bw4*80I* allele at the *HLA-A* locus

^6^  ● indicate the subjects who are Bw6 homozygous and have no *HLA-Bw4* alleles at the *HLA-A* locus.
